# Impacts of urban land-use change on land-surface heat exposure risk: spatial evidence from multi-source remote sensing in Zhejiang Province

**DOI:** 10.3389/fpubh.2026.1909236

**Published:** 2026-08-14

**Authors:** Yuansheng Huang, Weixiao Zhang, Mengmeng Shi, Aisulu Bayalieva, Gulmira Karabalaeva

**Affiliations:** 1 School of Art Design, Zhejiang Guangsha Vocational and Technical University of Construction, Dongyang, China; 2 Kyrgyz State University named after I. Arabaev, Bishkek, Kyrgyzstan; 3 School of Art and Design, Jiangxi Institute of Fashion Technology, Nanchang, China; 4 The Design School, Faculty of Innovation & Technology, Taylor's University, Subang Jaya, Malaysia; 5 Jusup Balasagyn Kyrgyz National University, Bishkek, Kyrgyzstan

**Keywords:** heat exposure, impervious-surface expansion, land surface temperature, land-surface heat exposure risk, land-use change, public heat-risk proxy

## Abstract

Rapid urbanization can intensify land-surface heat exposure by replacing natural surfaces with impervious materials, yet the thermal effects of specific land-cover transitions remain insufficiently understood. We integrated land-cover data for 2000, 2010, and 2020 with summer daytime land surface temperature (LST) and Normalized Difference Vegetation Index (NDVI) data for 2001, 2010, and 2020 to examine land-use change and LST-derived heat exposure across Zhejiang Province, China. Land-cover transition analysis, historical percentile-based heat-exposure identification, and regression models were applied. From 2000 to 2020, impervious surfaces expanded by 137.94%, with cropland accounting for 86.10% of newly added impervious surfaces. Mean summer daytime LST increased from 28.59 to 30.47 °C, while the 95th percentile increased from 31.45 to 36.09 °C. Newly added impervious surfaces exhibited the greatest warming (4.57 °C) and highest heat-exposure ratio (90.36%), whereas stable forestland showed the lowest warming (1.18 °C) and exposure ratio (9.24%). At the prefecture level, newly added impervious surfaces were positively associated with heat exposure (*R*^2^ = 0.69), while NDVI was negatively associated with LST warming (*R*^2^ = 0.81). Impervious-surface expansion and ecological buffering jointly shaped land-surface heat-exposure risk. Limiting heat-intensive land conversion and strengthening blue-green infrastructure in newly urbanized areas can support climate-adaptive spatial planning. These results represent an LST-derived public heat-risk proxy rather than directly observed health outcomes.

## 1 Introduction

Rapid urbanization is a major land-surface process that reshapes regional land-surface heat-exposure risk. With the expansion of construction land, roads, rooftops, plazas, and other hardened surfaces replace cropland, forestland, water bodies, wetlands, and other natural or semi-natural surfaces, thereby altering land-surface structure, surface energy balance, and ecological-regulation capacity (1–4). Classical urban-climate theory suggests that the albedo, heat capacity, roughness, soil moisture, evapotranspiration processes, and anthropogenic heat emissions of urban underlying surfaces jointly affect local thermal environments (5–7). When natural surfaces with relatively low heat-storage capacity and strong evapotranspiration are converted to impervious surfaces with stronger heat absorption and heat storage, latent heat flux tends to decrease, while sensible heat flux and heat storage increase. These changes can raise land surface temperature (LST) and expand areas exposed to high land-surface heat (1, 8–10). Therefore, land-use transition provides a direct entry point for explaining how heat-exposure spaces are produced during urbanization.

The public-health relevance of heat exposure has attracted increasing attention under climate change and rapid urban expansion (7, 11–13). Extreme heat can increase heat stress and may aggravate cardiovascular disease, respiratory disease, chronic illness burdens, outdoor-work risks, energy demand, sleep quality, and infrastructure operation (14–17). However, because this study does not use morbidity, mortality, or population-health records, the risk terminology is used carefully. Specifically, expressions such as “potential public heat risk,” “public heat-risk proxy,” and “land-surface heat-exposure risk” refer to LST-derived exposure conditions with public-health relevance rather than directly observed health outcomes. This distinction is important for ensuring that remote-sensing-based heat-exposure analysis is not overinterpreted as epidemiological evidence.

Remote sensing provides essential support for identifying the spatial linkage between land-use change and land-surface heat exposure (3, 5). Thermal-infrared remote sensing can characterize LST patterns; vegetation indices such as NDVI can reflect surface greenness and evapotranspiration potential; and land-cover products can identify the conversion of natural or semi-natural surfaces into artificial surfaces (17–20). Previous studies have shown that impervious-surface expansion is generally associated with higher LST, whereas vegetation cover, tree canopy, and blue–green spaces can reduce local temperatures through shading, evapotranspiration, and changes in surface thermophysical properties (21–25). Nevertheless, many studies still describe heat patterns without explicitly tracing how specific land-cover transitions reshape heat-exposure spaces.

Three research gaps remain particularly relevant. First, many studies focus on single-year LST patterns or urban heat-island intensity, with less attention to how long-term land-cover transition cumulatively changes heat-exposure risk. Second, although LST, NDVI, and land-cover data are often used together, the analysis is frequently dominated by correlation tests rather than mechanism-oriented land-change groups. Distinguishing newly added impervious surfaces, stable impervious surfaces, stable cropland, stable forestland, and stable water bodies can more directly reveal how cropland and forestland conversion to impervious surface is associated with warming and heat exposure. Third, in the absence of high-resolution health-outcome data, remote-sensing-based studies should avoid equating LST directly with disease risk while still constructing transparent exposure proxy indicators with public-health relevance. It is therefore necessary to establish a reproducible analytical framework that links land-cover transition, thermal response, heat exposure, and public heat-risk proxy indicators.

Zhejiang Province provides a representative case for this research question. As an important province along China’s southeastern coast and the southern wing of the Yangtze River Delta, Zhejiang has experienced substantial urban expansion and land-cover restructuring over the past two decades. Its northern and northeastern plains have concentrated high-intensity urban construction and population activities, while the southwestern and south-central mountainous and hilly areas still retain extensive forestland and ecological space. Coastal plains, river networks, mountain forests, and densely built urban areas coexist within the same provincial territory, giving Zhejiang a clear ‘urbanization-ecological space’ gradient. This complex surface structure makes it suitable for identifying how impervious-surface expansion, especially conversion from cropland and forestland, is associated with surface warming and heat exposure, while also allowing the buffering effects of vegetation cover and stable ecological spaces to be assessed.

Against this background, this study takes Zhejiang Province as the study area and develops a remote-sensing analytical framework of ‘land-use change-thermal-environment response-heat exposure-public heat-risk proxy’ to systematically evaluate land-cover change from 2000 to 2020 and its influence on summer daytime land-surface heat exposure from 2001 to 2020. Specifically, this study seeks to answer four questions: (1) How did the land-cover structure and the sources of impervious-surface expansion change in Zhejiang Province from 2000 to 2020? (2) Did summer daytime LST from 2001 to 2020 exhibit both overall warming and high-temperature-tail expansion? (3) Did newly added impervious surfaces, stable impervious surfaces, stable cropland, stable forestland, and stable water bodies show different warming magnitudes and heat-exposure ratios? (4) At the prefecture-level city scale, are newly added impervious-surface expansion and NDVI associated with the composite public heat-risk proxy index? This study helps extend land-use change research from conventional spatial-pattern analysis to heat-exposure-risk governance and provides scientific evidence for climate adaptation, territorial spatial regulation, and heat-exposure prevention in rapidly urbanizing regions.

## 2 Data and methods

### 2.1 Study area

**Figure 1:**
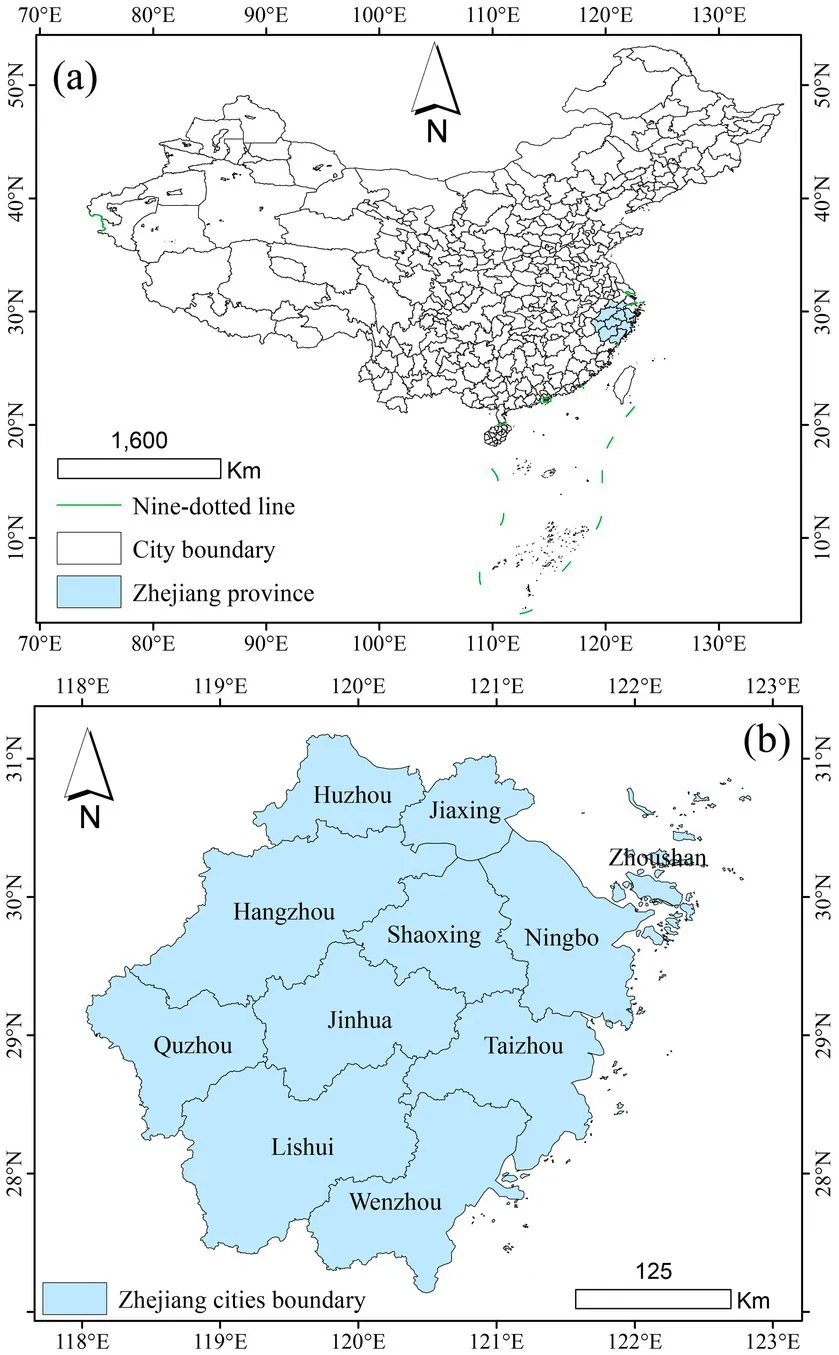
Location of the study area and prefecture-level administrative units. **(a)** Location of Zhejiang Province in China **(b)** Spatial distribution of the 11 prefecture-level cities in Zhejiang Province.

Zhejiang Province is located on the southeastern coast of China and the southern wing of the Yangtze River Delta, roughly between 27°N-31°N and 118°E-123°E. It is one of the eastern coastal provinces of China with a high level of urbanization and active land-use change. The study area borders the East China Sea to the east, Shanghai and Jiangsu to the north, Anhui and Jiangxi to the west, and Fujian to the south, forming a pronounced composite geographic gradient from coast to inland and from plains to mountains. This study covers the entire territory of Zhejiang Province, including 11 prefecture-level cities: Hangzhou, Ningbo, Wenzhou, Jiaxing, Huzhou, Shaoxing, Jinhua, Quzhou, Zhoushan, Taizhou, and Lishui (Figure 1). Zhejiang has a complex terrain pattern. Mountainous and hilly areas are widely distributed in the southwest and south-central regions, whereas plains, river networks, and coastal lowlands are relatively concentrated in the north and northeast. Influenced by topography, transportation location, and economic-development conditions, construction land and population activities are mainly concentrated along Hangzhou Bay, the northern Zhejiang Plain, and urban clusters such as Ningbo-Shaoxing-Jiaxing. In contrast, Lishui, Quzhou, and parts of the mountainous areas in southern Zhejiang retain relatively high proportions of forestland and ecological space. This marked land-use gradient provides a typical setting for identifying urban expansion, ecological-space change, and their thermal-environment effects. From the perspective of public heat-risk research, Zhejiang Province is highly representative. On the one hand, rapid urbanization has promoted continuous impervious-surface expansion, causing cropland, forestland, and other natural or semi-natural surfaces to be converted into construction land, thereby altering surface energy balance and summer thermal-environment patterns. On the other hand, pronounced differences in terrain, vegetation cover, and urbanization intensity within Zhejiang lead to substantial spatial heterogeneity in warming magnitude, heat exposure, and ecological buffering capacity across prefecture-level cities. Therefore, selecting Zhejiang Province as the study area helps reveal how land-use change affects potential public heat risk through impervious-surface expansion and vegetation-buffering change, and provides empirical evidence for heat-risk governance and territorial spatial optimization in rapidly urbanizing coastal regions of eastern China.

### 2.2 Remote-sensing data

Land-cover data were obtained from the China Land Cover Dataset (CLCD) v01 annual 30 m land-cover dataset. CLCD is constructed from large-scale Landsat imagery and Google Earth Engine and provides multi-year land-cover information for China. Its classification scheme includes cropland, forest, shrubland, grassland, water, snow/ice, barren land, impervious surface, and wetland. This study selected data for 2000, 2010, and 2020 to characterize land-cover change over two decades. Land surface temperature (LST) data were derived from the Moderate Resolution Imaging Spectroradiometer (MODIS) MOD11A2 v061 eight-day composite daytime LST product, with the temporal window set to June–August in 2001, 2010, and 2020. Vegetation data were obtained from the MODIS MOD13Q1 v061 16-day composite Normalized Difference Vegetation Index (NDVI) product, with the temporal window consistent with that of the LST data. Because MODIS data have a relatively coarse spatial resolution, they were used to analyze regional-scale heat-exposure patterns and change trends, rather than street-block-scale microclimates.

### 2.3 Research framework

**Figure 2:**
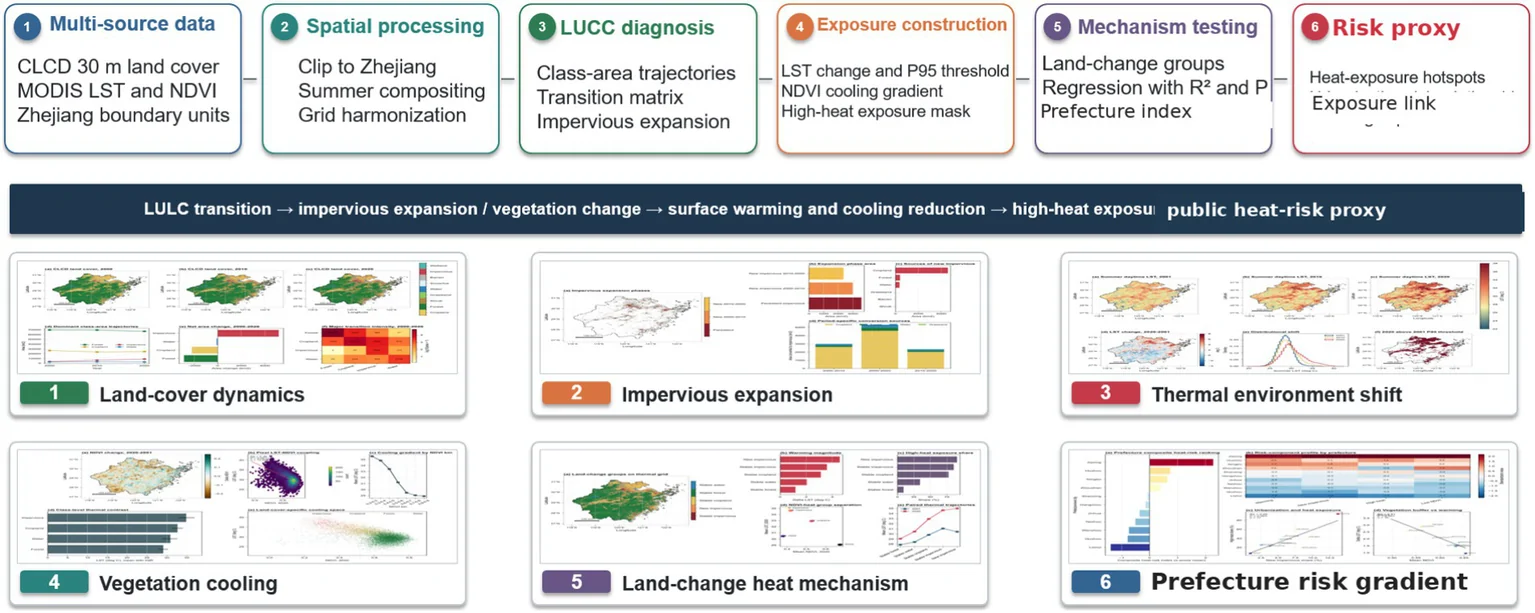
Research framework.

This study aims to reveal the land-surface processes through which land-use change contributes to LST-derived heat exposure and potential public heat risk. The overall analytical framework consists of six steps: multi-source data acquisition, spatial preprocessing, land-use change diagnosis, heat-exposure indicator construction, mechanism testing, and public heat-risk proxy inference (Figure 2). First, CLCD 30 m land-cover data, MODIS summer daytime LST and NDVI data, and administrative-boundary data for Zhejiang Province were integrated. Second, all spatial data were clipped to the study area, composited for the summer season, and harmonized to a common spatial grid. Third, land-cover change, impervious-surface expansion, and the source structure of expansion during 2000–2020 were identified. Fourth, LST change, heat exposure, and vegetation-cooling indicators were constructed. Finally, based on land-change types and prefecture-level city statistics, the relationships among urban expansion, vegetation cover, and the public heat-risk proxy were evaluated. This framework forms an analytical chain of ‘land-cover transition → impervious-surface expansion/vegetation change → surface warming and weakened cooling capacity → heat exposure → public heat-risk proxy indicators’ (26–28).

### 2.4 Data sources and spatial preprocessing

This study mainly used three types of data. The first type was CLCD land-cover data for 2000, 2010, and 2020, which were used to identify changes in land-use structure, land-cover transitions, and impervious-surface expansion (28–31). CLCD land-cover types include cropland, forestland, shrubland, grassland, water, snow/ice, barren land, impervious surface, and wetland; among them, impervious surface was used to represent the expansion of construction land. The second type was MODIS summer daytime LST and NDVI data for June–August in 2001, 2010, and 2020, which were used to represent the summer land-surface thermal environment and vegetation cover, respectively. The third type consisted of the provincial boundary of Zhejiang and the administrative boundaries of its 11 prefecture-level cities, which were used for study-area clipping, spatial masking, and prefecture-level city statistics. The original MODIS daytime LST values were converted to degrees Celsius using the scale factor, as shown in Equation 1 (32–34):

$$\mathrm{LS}T_{y,p}=DN_{y,p}^{\mathrm{LST}}*0.02-273.15$$ (1)

where $\mathrm{LS}T_{y,p}$ denotes the daytime land surface temperature of pixel *p* in year *y*, and $DN_{y,p}^{\mathrm{LST}}$ denotes the original MODIS LST pixel value.

The original NDVI values were converted using the MODIS scale factor, as shown in Equation 2 (12, 14, 15):

$$\mathrm{NDV}I_{y,p}=DN_{y,p}^{\text{NDVI}}*0.0001$$ (2)

where $\mathrm{NDV}I_{y,p}$ denotes the normalized difference vegetation index of pixel p in year y, and $DN_{y,p}^{\text{NDVI}}$ denotes the original MODIS NDVI pixel value.

For each year, all valid observations from June to August were averaged to obtain the summer mean LST and summer mean NDVI using Equation 3 (2, 3, 35, 36):

$${\bar{X}}_{y,p}=\frac{1}{n_{y,p}}\sum_{t=1}^{n_{y,p}}X_{y,p,t}$$ (3)

where ${\bar{X}}_{y,p}$ denotes the summer mean value of pixel *p* in year *y*, $X_{y,p,t}$ denotes the valid observation at time *t*, and $n_{y,p}$ denotes the number of valid summer observations. All raster data were clipped to the boundary of Zhejiang Province. Land-cover data retained their original categorical attributes in the land-use change analysis. When coupled with LST and NDVI, the CLCD data were resampled to the MODIS thermal-environment grid using the nearest-neighbor method to avoid altering categorical values through interpolation.

### 2.5 Diagnosis of land-use change

To characterize the evolution of land-use structure from 2000 to 2020, this study first calculated the area of each land-cover type in each year. The area of land-cover class *i* in year y was calculated using Equation 4 (37–39):

$$A_{i,y}=\sum_{p=1}^{N}I(L_{y,p}=i)*a_{p}$$ (4)

where $A_{i,y}$ denotes the area of land-cover class *i* in year *y*, $L_{y,p}$ denotes the land-cover type of pixel *p* in year *y*, I(.) is an indicator function that equals 1 when the condition is satisfied and 0 otherwise, and $a_{p}$ denotes the area of pixel *p*.

Furthermore, land-cover transition matrices for 2000–2010, 2010–2020, and 2000–2020 were constructed to identify the main conversion pathways among land types. The area converted from land type *i* to land type j was calculated using Equation 5 (40, 41):

$$T_{\mathrm{ij}}^{t_{1}\to t_{2}}=\sum_{p=1}^{N}I(L_{t_{1},p}=i,L_{t_{2},p}=j)*a_{p}$$ (5)

where denotes the area converted from land type i in year to land type j in year. This matrix was used to identify the main conversion relationships among cropland, forestland, water bodies, and impervious surfaces, and to reveal the land-use basis for the formation of land-surface heat-exposure risk.

### 2.6 Identification of impervious-surface expansion stages and sources

Impervious-surface expansion is the core spatial manifestation of land urbanization and an important land-use basis for elevated land-surface heat exposure (42–44). Based on CLCD data for 2000, 2010, and 2020, impervious surfaces were divided into three categories using Equations 6–8: persistent impervious surfaces, newly added impervious surfaces from 2000 to 2010, and newly added impervious surfaces from 2010 to 2020:

$$P_{p}^{\text{stable}}=I(L_{2000,p}=8,L_{2020,p}=8,)$$ (6)

$$P_{p}^{00-10}=I(L_{2000,p}\neq 8,L_{2010,p}=8,)$$ (7)

$$P_{p}^{10-20}=I(L_{2010,p}\neq 8,L_{2020,p}=8,)$$ (8)

In this context, code 8 represents an impervious surface. Represents a persistent impervious surface, represents newly added impervious surfaces from 2000 to 2010, and represents newly added impervious surfaces from 2010 to 2020.

To further identify the sources of newly added impervious surfaces, this study calculated the areas converted from different land-cover types to impervious surfaces in each period using Equation 9:

$$S_{k}^{t_{1}\to t_{2}}=\sum_{p=1}^{N}I(L_{t_{1},p}=k,L_{t_{2},p}=8)*a_{p}$$ (9)

where denotes the area converted from land type k to impervious surface during the period from to. This indicator was used to determine whether newly added construction land mainly originated from cropland, forestland, water bodies, grassland, or other land types, thereby revealing the spatial sources through which land-use change contributes to heat-exposure expansion.

### 2.7 Construction of heat-exposure indicators

To characterize changes in the summer daytime land-surface thermal environment, this study calculated the summer mean LST for 2001, 2010, and 2020, and further calculated the magnitude of LST change from 2001 to 2020 using Equation 10 (31, 45, 46):

$$\mathrm{\Delta LS}T_{p}=\mathrm{LS}T_{2020,p}-\mathrm{LS}T_{2001,p}$$ (10)

where $\mathrm{\Delta LS}T_{p}$ denotes the warming magnitude of pixel p during 2001–2020. In addition to mean warming, this study further calculated the median, quartiles, and 95th percentile of LST in different years to identify whether the regional thermal environment experienced overall upward shifting and high-temperature-tail expansion.

Heat-exposure areas were identified using a historical relative-threshold method. The 95th percentile of summer daytime LST in 2001 was used as the historical high-temperature threshold (31, 41, 47, 48). This threshold should be interpreted as a land-surface exposure threshold rather than as a universal physiological or epidemiological health threshold. The historical high-temperature threshold was calculated using Equation 11:

$$T_{95}=P_{95}(\mathrm{LS}T_{2001})$$ (11)

where $T_{95}$ denotes the 95th percentile of the LST distribution in 2001. If the LST of a pixel in 2020 was greater than or equal to this threshold, the pixel was defined as a heat-exposure pixel using Equation 12:

$$H_{p}=\{\begin{array}{c} 1,\mathrm{LS}T_{2020,p}\geq T_{95}\\ 0,\mathrm{LS}T_{2020,p}<T_{95} \end{array}$$ (12)

where denotes the heat-exposure status of pixel p. This indicator was used to measure the spatial expansion of heat exposure in 2020 relative to the extreme high-temperature level at the beginning of the study period. Applying the same baseline threshold to 2020 enabled temporal comparability between baseline extreme conditions and later exposure patterns. It should be noted that the heat exposure defined in this study is an LST-derived public heat-risk proxy indicator and is not equivalent to directly observed morbidity, mortality, or human-health damage.

### 2.8 Vegetation cooling effect and LST-NDVI coupling analysis

To evaluate the buffering effect of vegetation-cover change on the thermal environment, this study first calculated NDVI change from 2001 to 2020 using Equation 13 (49–51):

$$\text{ΔNDV}I_{p}=\mathrm{NDV}I_{2020,p}-\mathrm{NDV}I_{2001,p}$$ (13)

where Delta $\text{ΔNDV}I_{p}$ denotes the vegetation-cover change of pixel *p*. Subsequently, a linear regression model between LST and NDVI was established using valid pixel samples in 2020, as specified in Equation 14:

$$\mathrm{LS}T_{2020,p}=\alpha +\text{βNDV}I_{2020,p}+\varepsilon_{p}$$ (14)

where α is the intercept, beta denotes the marginal effect of NDVI on LST, and $\varepsilon_{p}$ is the residual term. Model explanatory power was represented by *R*^2^, and statistical significance was tested using *p*-values. If β < 0, higher vegetation cover is associated with lower LST.

To further identify the cooling effect along the vegetation-cover gradient, NDVI was divided into intervals at a step of 0.1, and the mean LST in each NDVI interval was calculated using Equation 15:

$${\bar{\mathrm{LST}}}_{b}=\frac{1}{N_{b}}\sum_{p\in b}\mathrm{LS}T_{2020,p}$$ (15)

where ${\bar{\mathrm{LST}}}_{b}$ denotes the mean LST in NDVI interval b, and $N_{b}$ denotes the number of valid pixels in that interval. This analysis was used to identify gradients of LST change under different levels of vegetation cover. In addition, the mean LST, interquartile range, and mean NDVI in 2020 were calculated by land-cover type to compare thermal-environment differences among major land types such as impervious surfaces, cropland, forestland, and water bodies.

### 2.9 Mechanism testing of land-change-associated heat exposure

To assess the association between land-use change and LST-derived heat exposure, this study overlaid the land-cover change results with LST and NDVI data and constructed five land-change groups: stable impervious surfaces, newly added impervious surfaces, stable cropland, stable forestland, and stable water bodies (52–55). These groups were defined using Equations 16–20:

$$G_{p}^{\mathrm{SI}}=I(L_{2000,p}=8,L_{2020,p}=8)$$ (16)

$$G_{p}^{\mathrm{NI}}=I(L_{2000,p}\neq 8,L_{2020,p}=8)$$ (17)

$$G_{p}^{\mathrm{SC}}=I(L_{2000,p}=1,L_{2020,p}=1)$$ (18)

$$G_{p}^{\mathrm{SF}}=I(L_{2000,p}=2,L_{2020,p}=2)$$ (19)

$$G_{p}^{\mathrm{SW}}=I(L_{2000,p}=5,L_{2020,p}=5)$$ (20)

where $G_{p}^{\mathrm{SI}}$ , $G_{p}^{\mathrm{NI}}$ , $G_{p}^{\mathrm{SC}}$ , $G_{p}^{\mathrm{SF}}$ , and $G_{p}^{\mathrm{SW}}$ denote stable impervious surfaces, newly added impervious surfaces, stable cropland, stable forestland, and stable water bodies, respectively.

For each land-change group, the mean LST in 2001, the mean LST in 2020, the mean warming magnitude from 2001 to 2020, the mean NDVI in 2020, and the heat-exposure ratio in 2020 were calculated (56–58). The mean warming magnitude for land-change group g was calculated using Equation 21:

$${\bar{\text{ΔLST}}}_{g}=\frac{1}{N_{g}}\sum_{p\in b}(\mathrm{LS}T_{2020,p}-\mathrm{LS}T_{2001,p})$$ (21)

where ${\bar{\text{ΔLST}}}_{g}$ denotes the mean warming magnitude of land-change group g, and $N_{g}$ denotes the number of valid pixels in that group.

The heat-exposure ratio of land-change group *g* was calculated using Equation 22:

$$E_{g}=\frac{\sum_{p\in g}H_{p}}{N_{g}}*100\%$$ (22)

where denotes the heat-exposure ratio within land-change group g. By comparing warming magnitudes and heat-exposure ratios among land-change types, this study identified how different land-use processes were associated with higher or lower LST-derived heat exposure.

### 2.10 Construction of a prefecture-level public heat-risk proxy index

To evaluate regional differentiation in the LST-derived public heat-risk proxy, this study constructed a composite heat-risk proxy index at the prefecture-level city scale. The index comprises four risk components: LST warming magnitude, proportion of newly added impervious surface, heat-exposure ratio, and low-NDVI risk (59–62). Among them, LST warming magnitude reflects the deterioration of the thermal environment; the proportion of newly added impervious surface reflects land-urbanization intensity; the heat-exposure ratio reflects the spatial extent of potential heat exposure; and low-NDVI risk reflects insufficient vegetation-buffering capacity.

For any prefecture-level city u, the standardized value of indicator X was calculated using Equation 23:

$$Z_{X,u}=\frac{X_{u}-\mu_{X}}{\sigma_{X}}$$ (23)

where $X_{u}$ denotes the standardized value of a given risk indicator for prefecture-level city u, and $\mu_{X}$ and $\sigma_{X}$ denote the mean and standard deviation of that indicator across all prefecture-level cities, respectively.

Because lower NDVI generally indicates weaker ecological-buffering capacity, NDVI was converted into a low-NDVI risk term using Equation 24:

$$Z_{\text{LowNDVI},u}=-\frac{\mathrm{NDV}I_{u}-\text{μNDVI}}{\text{σNDVI}}$$ (24)

Finally, the composite public heat-risk proxy index for each prefecture-level city was calculated using Equation 25:

$$\mathrm{HR}I_{u}=\frac{Z_{\text{ΔLST},u}+Z_{\text{NewImp},u}+Z_{\text{HighHeat},u}+Z_{\text{LowNDVI},u}}{4}$$ (25)

where denotes the composite public heat-risk proxy index of prefecture-level city u;, and denote the standardized warming magnitude, standardized proportion of newly added impervious surface, standardized heat-exposure ratio, and standardized low-NDVI risk, respectively. A higher indicates stronger potential public heat risk arising from the combined effects of land urbanization, thermal-environment deterioration, heat exposure, and insufficient vegetation buffering.

Equal weighting was adopted as a transparent exploratory strategy to avoid introducing subjective priority among the four risk dimensions, rather than to prescribe an externally calibrated health-risk weighting structure.

Furthermore, two prefecture-level linear regression models were established to test the associations of land urbanization and vegetation cover with the LST-derived public heat-risk proxy, as specified in Equation 26 and Equation 27, respectively:

$$\text{HighHea}t_{u}=\alpha_{1}+\beta_{1}\text{NewIm}p_{u}+\varepsilon_{1,u}$$ (26)

$$\mathrm{\Delta LS}T_{u}=\alpha_{2}+\beta_{2}\mathrm{NDV}I_{u}+\varepsilon_{2,u}$$ (27)

where $\text{HighHea}t_{u}$ denotes the heat-exposure ratio of prefecture-level city *u*, $\text{NewIm}p_{u}$ denotes the proportion of newly added impervious surface, Delta LST_u denotes the mean warming magnitude, and $\mathrm{NDV}I_{u}$ denotes the mean vegetation-cover level. The first model was used to test whether newly added impervious-surface expansion increased the heat-exposure ratio, and the second model was used to test whether vegetation cover buffered surface warming. The explanatory power and statistical significance of both models were evaluated using *R*^2^ and *p*-values.

## 3 Results

### 3.1 Evolution of land-cover patterns and transition characteristics

**Figure 3:**
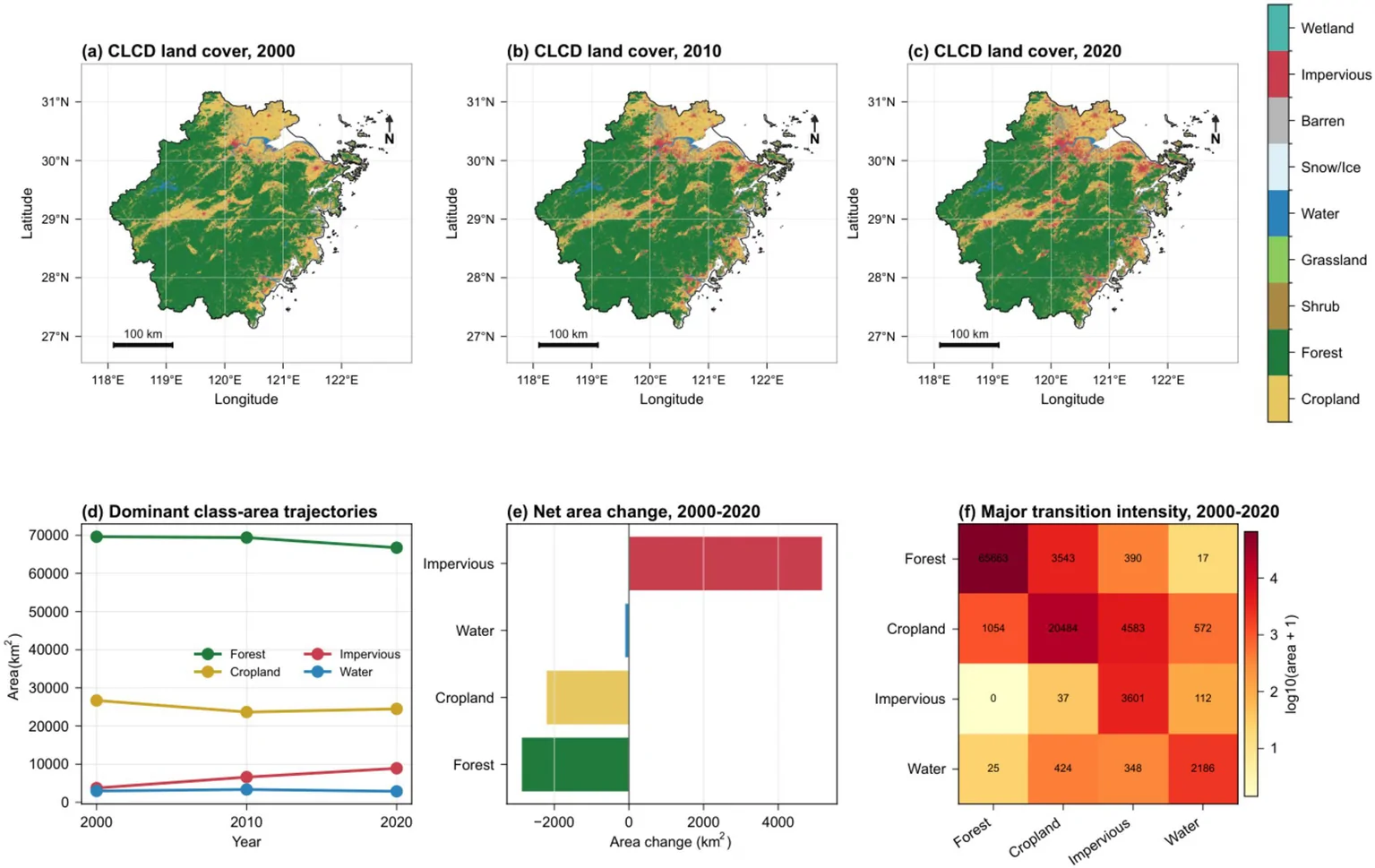
Evolution of land-cover patterns and major transition characteristics in the study area from 2000 to 2020. **(a–c)** Spatial patterns of CLCD land cover in 2000, 2010, and 2020, respectively; **(d)** Area-change trajectories of major land-cover types; **(e)** Net area changes of major land-cover types from 2000 to 2020; **(f)** Transition-intensity matrix of major land-cover types from 2000 to 2020.

From 2000 to 2020, the land-cover pattern of the study area was generally characterized by the dominance of forestland and cropland, accompanied by continuous impervious-surface expansion (Figure 3). Spatially, forestland was mainly distributed in the western, southern, and central mountainous and hilly areas of the study region, forming the main body of regional ecological space. Cropland was concentrated mainly in the northern plains, river valleys, and areas surrounding urban settlements. Impervious surfaces, which were relatively scattered in 2000, gradually expanded toward urban cores, transportation corridors, and densely built coastal urban areas, showing a clear trend of urban expansion (Figures 3a–c). Area statistics show that forestland was consistently the largest land-cover type in the study area, but its area decreased from 69,616.69 km^2^ in 2000 to 66,750.85 km^2^ in 2020, a net reduction of 2,865.83 km^2^, with its proportion declining from 67.54 to 64.76% (Figures 3d,e). Cropland decreased from 26,695.27 km^2^ in 2000 to 23,663.26 km^2^ in 2010, and then rebounded to 24,493.30 km^2^ in 2020; nevertheless, it remained 2,201.97 km^2^ lower than in 2000. In contrast to the declines in forestland and cropland, impervious surfaces continued to increase, from 3,750.74 km^2^ in 2000 to 8,924.35 km^2^ in 2020, a net increase of 5,173.61 km^2^ and a growth rate of 137.94%. The water area changed only slightly, decreasing from 2,984.49 km^2^ in 2000 to 2,887.77 km^2^ in 2020, indicating that it remained generally stable during the study period. Land-cover transition results further show that the major land-cover types in the study area had a certain degree of stability, although local conversion processes were pronounced (Figure 3f). From 2000 to 2020, the areas of forestland, cropland, impervious surface, and water bodies that remained unchanged were 65,662.81 km^2^, 20,484.46 km^2^, 3,601.33 km^2^, and 2,186.12 km^2^, respectively, indicating that the basic regional land-cover structure did not undergo fundamental change. However, among the main transition pathways, conversion from cropland to impervious surface was the most prominent, reaching 4,583.06 km^2^, and represented the primary source of impervious-surface expansion. In addition, the conversion from forestland to cropland covered 3,542.74 km^2^, while the conversion from cropland to forestland covered 1,053.72 km^2^, indicating a strong bidirectional conversion between forestland and cropland. Overall, land-cover change in the study area from 2000 to 2020 was mainly characterized by rapid impervious-surface expansion and the simultaneous contraction of forestland and cropland, with the conversion of cropland to impervious surface being the dominant land-transition process.

### 3.2 Stages and source structure of impervious-surface expansion

**Figure 4:**
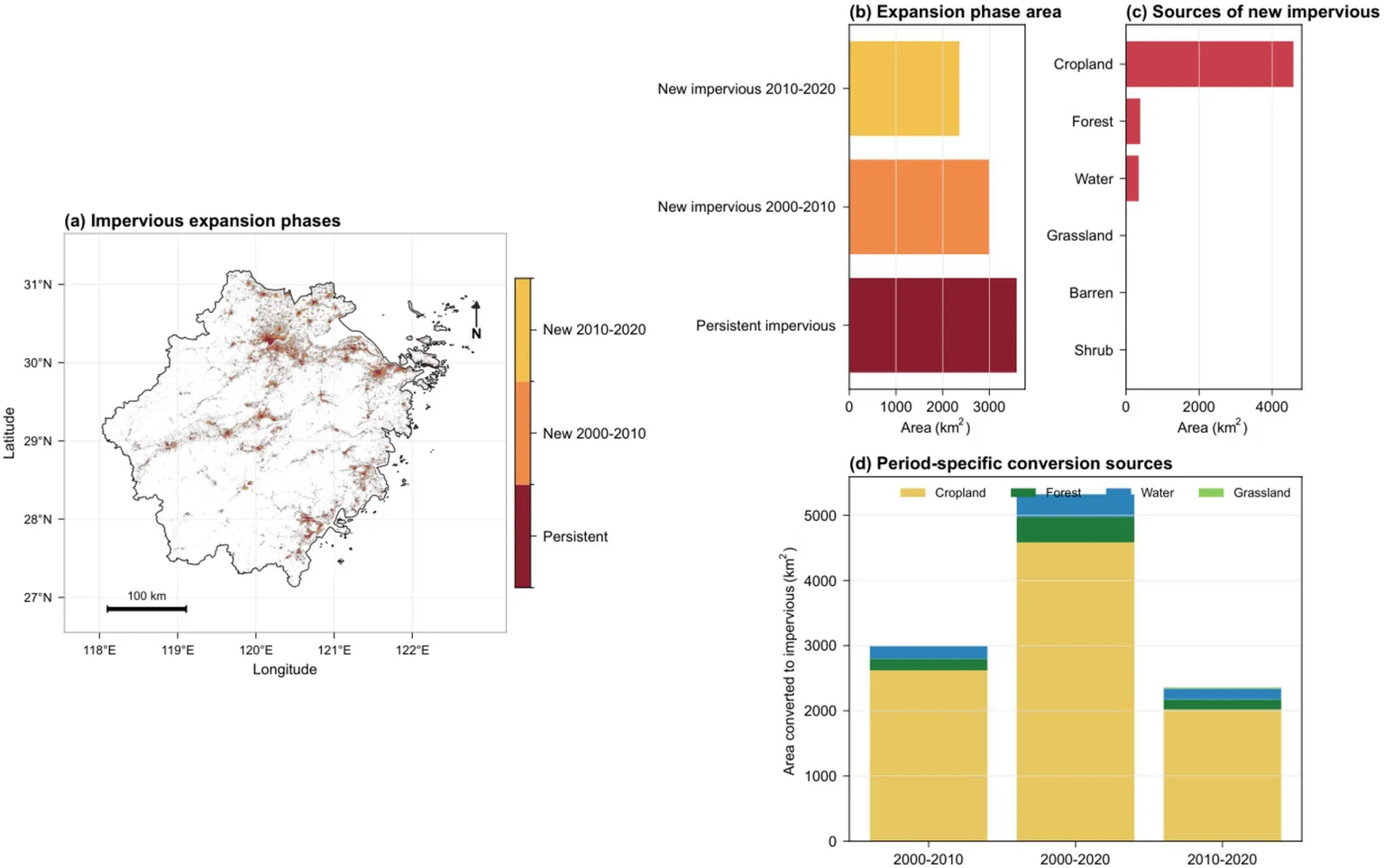
Stages and source structure of impervious-surface expansion in the study area from 2000 to 2020: **(a)** Spatial distribution of impervious-surface expansion stages; **(b)** Areas of persistent impervious surfaces and newly added impervious surfaces in different periods; **(c)** Land-cover sources of newly added impervious surfaces from 2000 to 2020; **(d)** Source composition of newly added impervious surfaces in different periods.

The stage-based identification of impervious-surface expansion indicates that construction-land growth in the study area from 2000 to 2020 had a clear temporal cumulative pattern (Figure 4). Spatially, persistent impervious surfaces were mainly concentrated in and around existing urban cores, whereas newly added impervious surfaces expanded outward along urban fringes, transportation corridors, and densely built coastal urban areas, forming a spatial pattern that spread from core agglomerations to peripheral areas (Figure 4a). In terms of area composition, among impervious surfaces in 2020, persistent impervious surfaces covered 3,588.58 km^2^, accounting for 40.12%; newly added impervious surfaces during 2000–2010 covered 2,995.98 km^2^, accounting for 33.50%; and newly added impervious surfaces during 2010–2020 covered 2,359.80 km^2^, accounting for 26.38% (Figure 4b). This indicates that impervious-surface expansion in the study area occurred mainly after 2000, with stronger expansion intensity during 2000–2010 than during 2010–2020. The source structure of newly added impervious surfaces shows that cropland was the dominant source of construction-land expansion (Figures 4c,d). From 2000 to 2020, the area converted from cropland to impervious surface reached 4,584.15 km^2^, accounting for 86.10% of all newly added impervious surfaces. Forestland and water bodies contributed 389.65 km^2^ and 348.18 km^2^, respectively, accounting for 7.32 and 6.54%; grassland, barren land, and shrubland made only minor contributions. By stage, the area converted from cropland to impervious surface during 2000–2010 was 2,621.23 km^2^, accounting for 87.49% of the newly added impervious-surface sources in that period. During 2010–2020, the cropland contribution decreased to 2,021.08 km^2^, but its share remained 85.65%. Therefore, although impervious-surface expansion slowed in the latter decade, its source structure did not change fundamentally, and cropland continued to serve as the primary spatial supply for construction-land expansion.

### 3.3 Increase in summer daytime land surface temperature and distributional shift of the thermal environment

**Figure 5:**
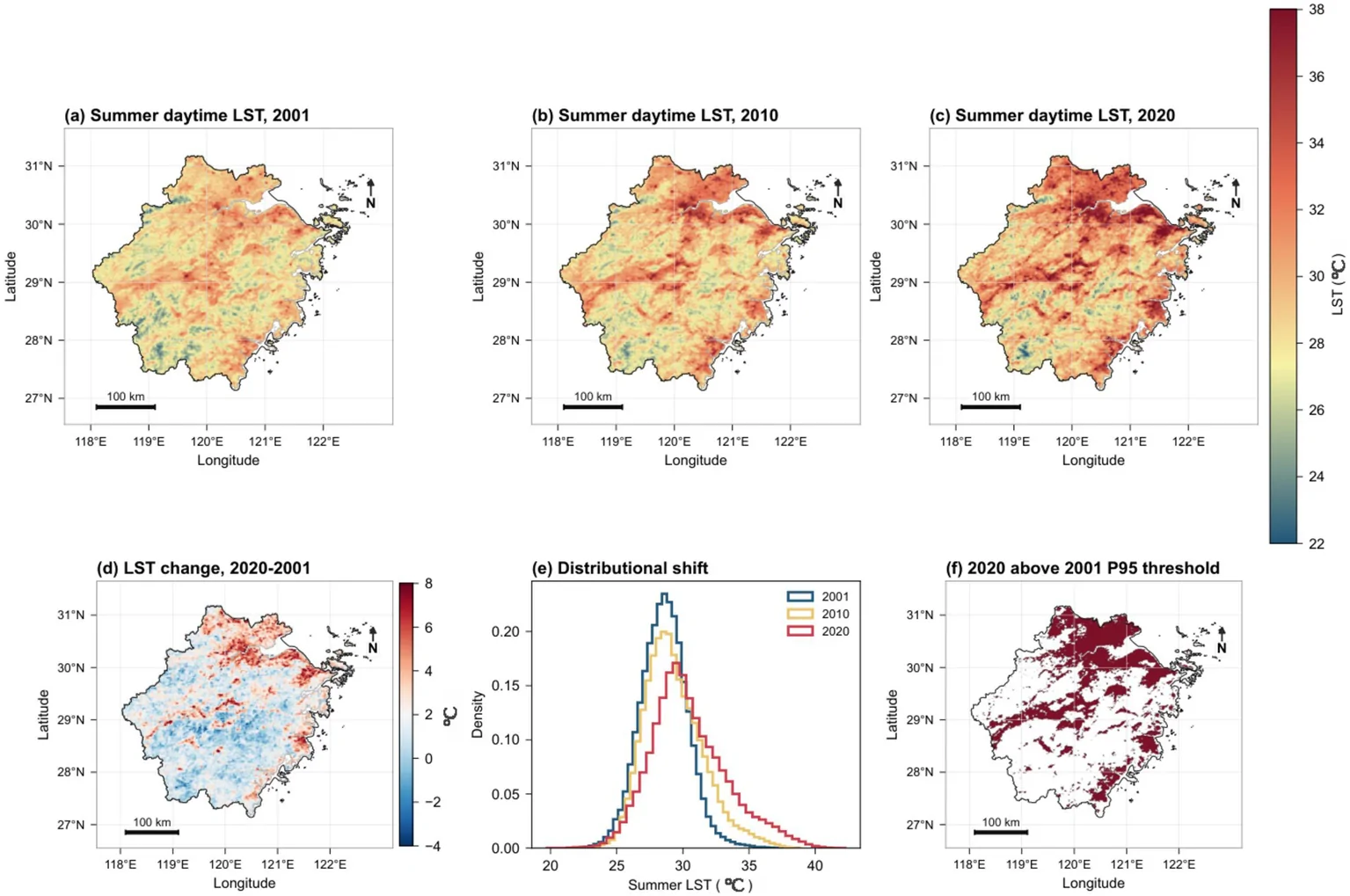
Changes in summer daytime land surface temperature and distributional shift of the thermal environment in Zhejiang Province from 2001 to 2020, derived from the MODIS MOD11A2 v061 summer daytime LST product at the MODIS thermal-grid scale. **(a–c)** Spatial distributions of summer daytime LST in 2001, 2010, and 2020, respectively; **(d)** LST change magnitude from 2001 to 2020; **(e)** LST distribution curves for the three study years; and **(f)** areas in 2020 exceeding the 2001 95th-percentile LST threshold. The threshold is a relative historical benchmark for within-region temporal comparison rather than an absolute physiological or epidemiological health threshold.

From 2001 to 2020, summer daytime LST in the study area showed a clear increasing trend, accompanied by spatial expansion of high-temperature areas (Figure 5). In 2001, regional LST was generally at a relatively low level, and high-temperature areas were scattered mainly across the northern plains, urban agglomerations, and parts of the coastal region. By 2010, high-temperature patches had begun to expand toward urban peripheries and transportation corridors. By 2020, high-temperature areas had become more contiguous, particularly in the northern, northeastern, and central densely built urban areas (Figures 5a–c). In terms of the magnitude of change from 2001 to 2020, most areas showed warming, with stronger warming concentrated mainly in and around active urban-expansion zones, whereas some mountainous forestland and ecological spaces maintained relatively low warming magnitudes (Figure 5d). The LST distribution curves further show that the thermal environment of the study area was characterized not only by an increase in the mean but also by marked expansion of the high-temperature tail (Figure 5e). The regional mean LST increased from 28.59 °C in 2001 to 29.33 °C in 2010 and further to 30.47 °C in 2020, with a cumulative increase of 1.89 °C from 2001 to 2020. Over the same period, the median increased from 28.58 °C to 30.05 °C, indicating a systematic upward shift in the overall thermal-environment level. Notably, the increase at the upper end of the LST distribution was more pronounced: the 95th percentile rose from 31.45 °C in 2001 to 36.09 °C in 2020, an increase of 4.63 °C, indicating that the expansion intensity of extreme high-temperature areas exceeded the level of mean regional warming. Using the 95th percentile of LST in 2001 (31.45 °C) as the high-temperature reference threshold, areas exceeding this threshold in 2020 were widely distributed across the northern, central, and coastal urbanized regions of the study area, revealing a substantial expansion of summer daytime heat-exposure space (Figure 5f). This threshold represents a relative historical benchmark for comparing changes within Zhejiang Province and should not be interpreted as an absolute physiological or epidemiological health threshold.

### 3.4 Coupling between vegetation-cover change and summer daytime LST

**Figure 6:**
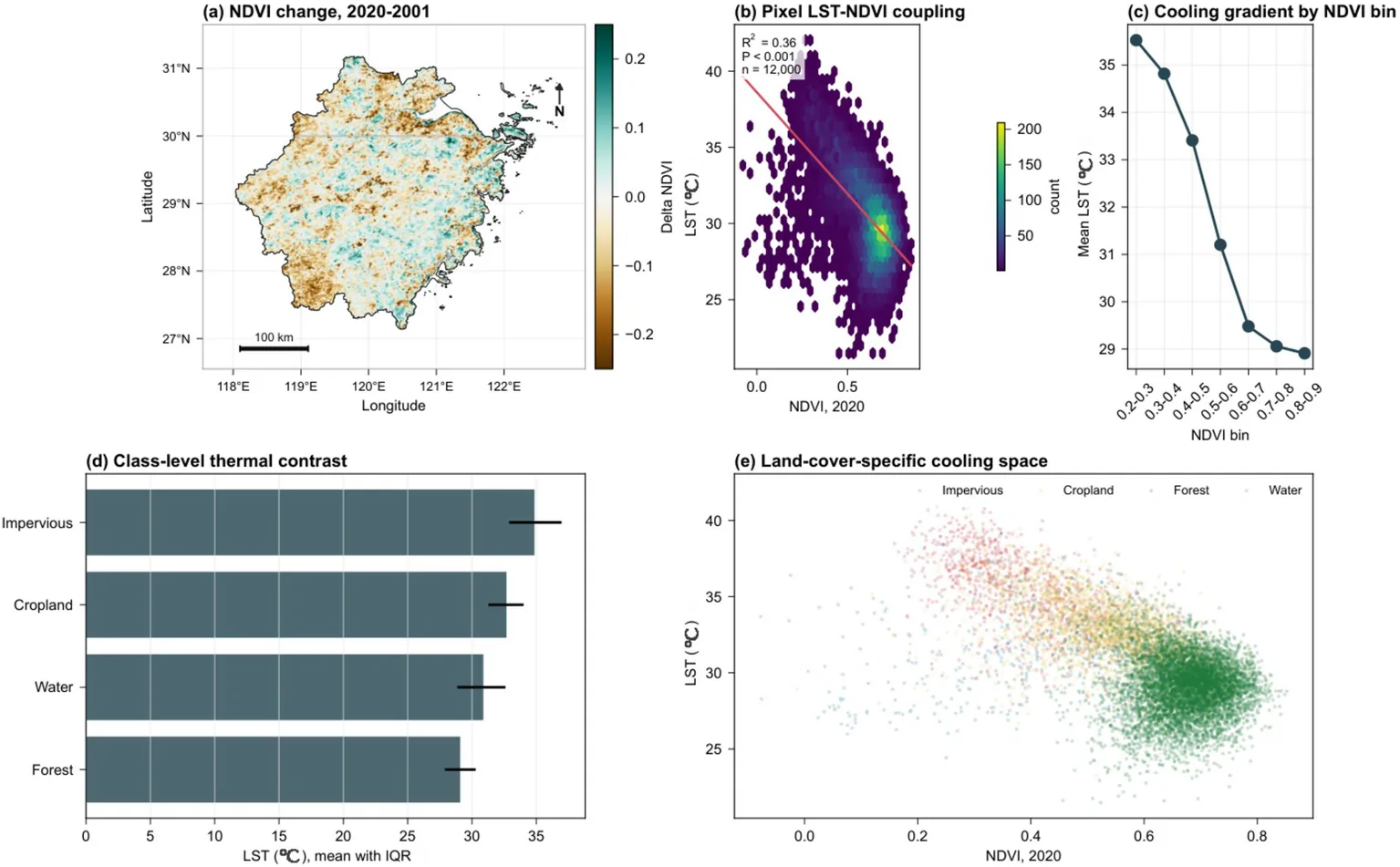
Coupling between vegetation cover and summer daytime land surface temperature in Zhejiang Province, based on MODIS MOD13Q1 v061 NDVI and MOD11A2 v061 daytime LST data harmonized to the MODIS thermal-grid scale. **(a)** Spatial distribution of NDVI change from 2001 to 2020; **(b)** pixel-scale relationship between NDVI and LST in 2020; **(c)** mean LST across 0.1-wide NDVI intervals; (d) mean LST and interquartile range by land-cover type; and **(e)** NDVI–LST distributions for the major land-cover types.

Vegetation-cover change showed a significant spatial coupling relationship with LST (Figure 6). From 2001 to 2020, NDVI change in the study area exhibited marked spatial heterogeneity. Areas with increased NDVI were mainly distributed in the central, eastern, and parts of the coastal regions, whereas the southwest and some urban-expansion areas experienced varying degrees of NDVI decline (Figure 6a). This spatial pattern indicates that vegetation cover did not improve synchronously across the entire study period, but was closely related to land-use change, urban expansion, and the distribution of ecological spaces. At the pixel scale in 2020, NDVI was significantly negatively correlated with LST (Figure 6b). The linear-fit results show that each unit increase in NDVI was associated with an average decrease of 13.32 °C in LST, with an explanatory power of *R*^2^ = 0.36, and the relationship passed the significance test (*p* < 0.001, *n* = 12,000). In other words, each 0.1 increase in NDVI corresponded to an approximate 1.33 °C decrease in LST, indicating that improved vegetation cover played a significant role in mitigating the summer daytime land-surface thermal environment. The mean LST across different NDVI intervals further revealed the gradient characteristics of the vegetation cooling effect (Figure 6c). When NDVI was in the 0.2–0.3 interval, mean LST reached 35.52 °C. As NDVI increased, LST declined continuously, reaching 28.91 °C in the 0.8–0.9 interval. The temperature difference between the two intervals was 6.61 °C, indicating that highly vegetated areas had a pronounced thermal-buffering effect. It is worth noting that the LST decrease was most evident when NDVI increased from 0.4 to 0.6, while the cooling magnitude tended to level off after NDVI exceeded 0.7, suggesting a possible marginally diminishing vegetation cooling effect. Differences in thermal environments among land-cover types further confirmed the cooling effect of vegetation cover (Figures 6d,e). In 2020, impervious surfaces had the highest mean LST, at 34.86 °C, followed by cropland at 32.68 °C, whereas water bodies and forestland had mean LST values of 30.88 °C and 29.09 °C, respectively. Compared with impervious surfaces, forestland, cropland, and water bodies had mean LST values lower by 5.77 °C, 2.18 °C, and 3.97 °C, respectively. The scatter distributions also show that forestland was concentrated mainly in the high-NDVI, low-LST range, whereas impervious surfaces were concentrated in the low-NDVI, high-LST range; cropland lay between the two, with moderate vegetation cover and moderately high temperature. Overall, increased vegetation cover substantially reduced summer daytime LST, and differences among land-cover types further reinforced the spatial differentiation of the regional thermal environment.

### 3.5 Newly added impervious surfaces as key land-surface units associated with elevated heat-exposure risk

**Figure 7:**
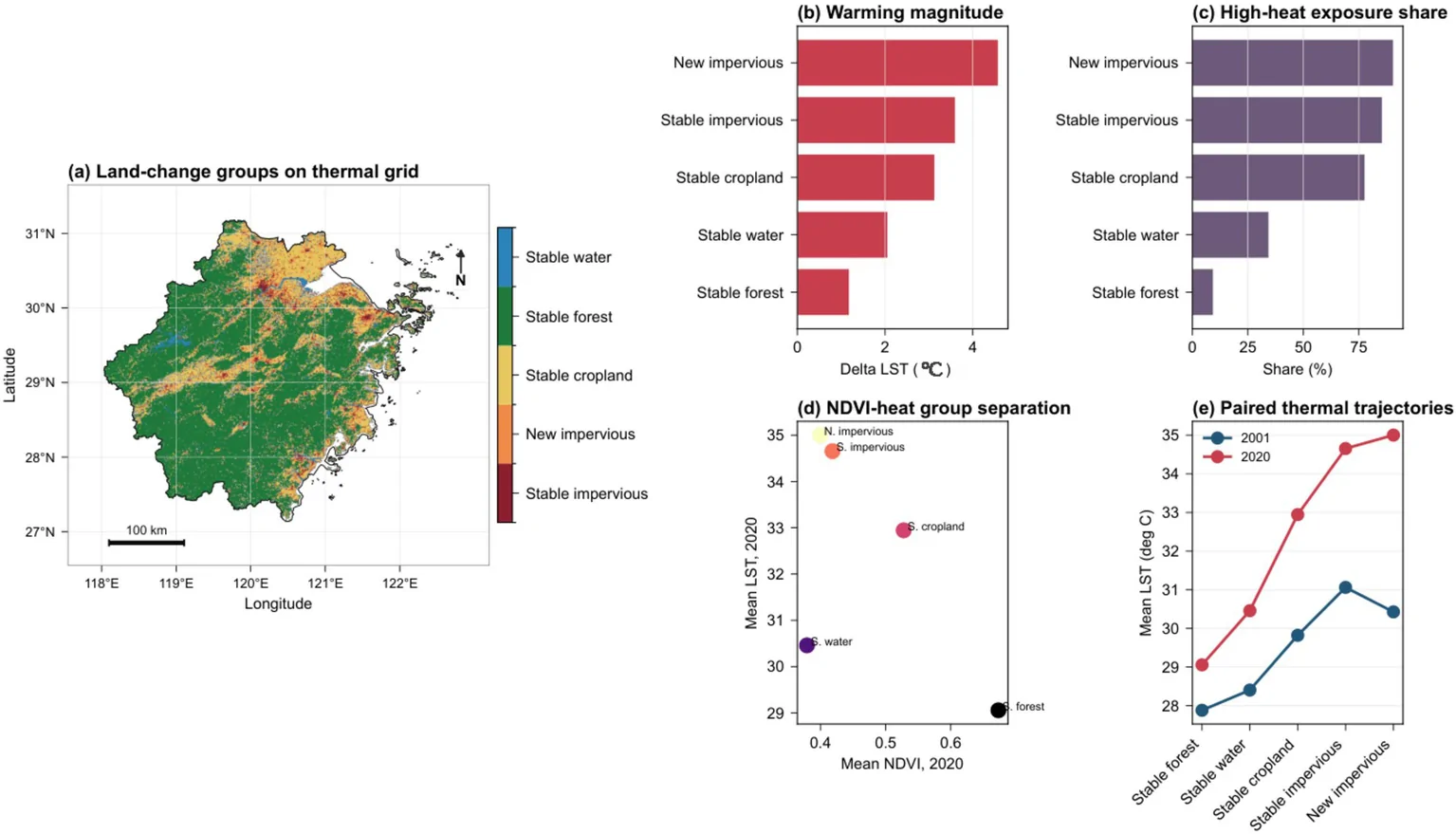
Thermal responses and heat-exposure characteristics of major land-change groups derived by overlaying CLCD 30 m land-cover transitions with MODIS LST and NDVI data on the harmonized MODIS thermal grid. **(a)** Spatial distribution of stable impervious surfaces, newly added impervious surfaces, stable cropland, stable forestland, and stable water bodies; **(b)** mean LST warming magnitude from 2001 to 2020; **(c)** proportion of pixels in each land-change group exceeding the 2001 95th-percentile LST threshold in 2020; **(d)** mean NDVI and mean LST in 2020 by land-change group; and **(e)** paired mean-LST trajectories between 2001 and 2020.

Different land-change types showed clearly differentiated thermal-environment responses (Figure 7). In terms of spatial distribution, stable forestland remained the dominant background type in the study region, widely distributed across the western, southern, and central mountainous and hilly areas. Stable cropland was mainly located in the northern plains and river valleys. Stable impervious surfaces and newly added impervious surfaces were concentrated around urban cores, densely built coastal urban areas, and transportation corridors (Figure 7a). This spatial pattern indicates that thermal-environment differences induced by land-cover change were concentrated mainly in areas of active urban expansion. In terms of warming magnitude from 2001 to 2020, newly added impervious surfaces experienced the most pronounced warming, with a mean LST increase of 4.57 °C. Stable impervious surfaces ranked second, with an increase of 3.59 °C, followed by stable cropland with an increase of 3.12 °C. Stable water bodies and stable forestland showed relatively low warming magnitudes, at 2.05 °C and 1.18 °C, respectively (Figure 7b). This indicates that impervious-surface expansion areas not only had higher temperature levels but also displayed stronger warming sensitivity over the long-term change process. By contrast, stable forestland had an obvious buffering effect on regional warming and represented the land-cover type with the lowest warming magnitude. The heat-exposure ratio further revealed differences in LST-derived heat-exposure risk among land-change types (Figure 7c). In 2020, the heat-exposure ratio of newly added impervious surfaces reached 90.36%, higher than that of stable impervious surfaces (85.36%) and stable cropland (77.55%). The heat-exposure ratio of stable water bodies was 34.23%, while that of stable forestland was only 9.24%. These results indicate that newly added impervious surfaces were the land-change type with the most concentrated heat exposure and showed substantially higher land-surface heat-exposure risk than stable natural or semi-natural surfaces. The NDVI-thermal-environment grouping results show that different land-change types were clearly separated in the ‘vegetation cover-LST’ space (Figure 7d). Stable forestland had the highest mean NDVI (0.67) and the lowest mean LST in 2020 (29.06 °C), whereas newly added impervious surfaces and stable impervious surfaces had lower NDVI and mean LST values of 35.00 °C and 34.66 °C, respectively. Stable cropland lay between these types, with moderate NDVI and relatively high LST. Although water bodies had relatively low NDVI, their mean LST was still markedly lower than that of impervious surfaces, indicating that in addition to vegetation cover, the thermophysical properties of the surface type itself also affect the regional thermal environment. Paired thermal-environment trajectories further show that all land-change types warmed from 2001 to 2020, but the magnitude of warming differed clearly (Figure 7e). In 2001, LST differences among types were already evident, with stable forestland being the coolest and stable impervious surfaces the warmest. By 2020, newly added impervious surfaces had become the hottest type, and the mean LST difference between them and stable forestland had widened to 5.94 °C. Overall, land urbanization conversion was associated with intensified local warming and heat exposure, whereas stable forestland showed the strongest regulatory effect in mitigating the deterioration of the regional thermal environment.

### 3.6 Prefecture-level differentiation of land-surface heat-exposure risk and its urbanization-vegetation controls

**Figure 8:**
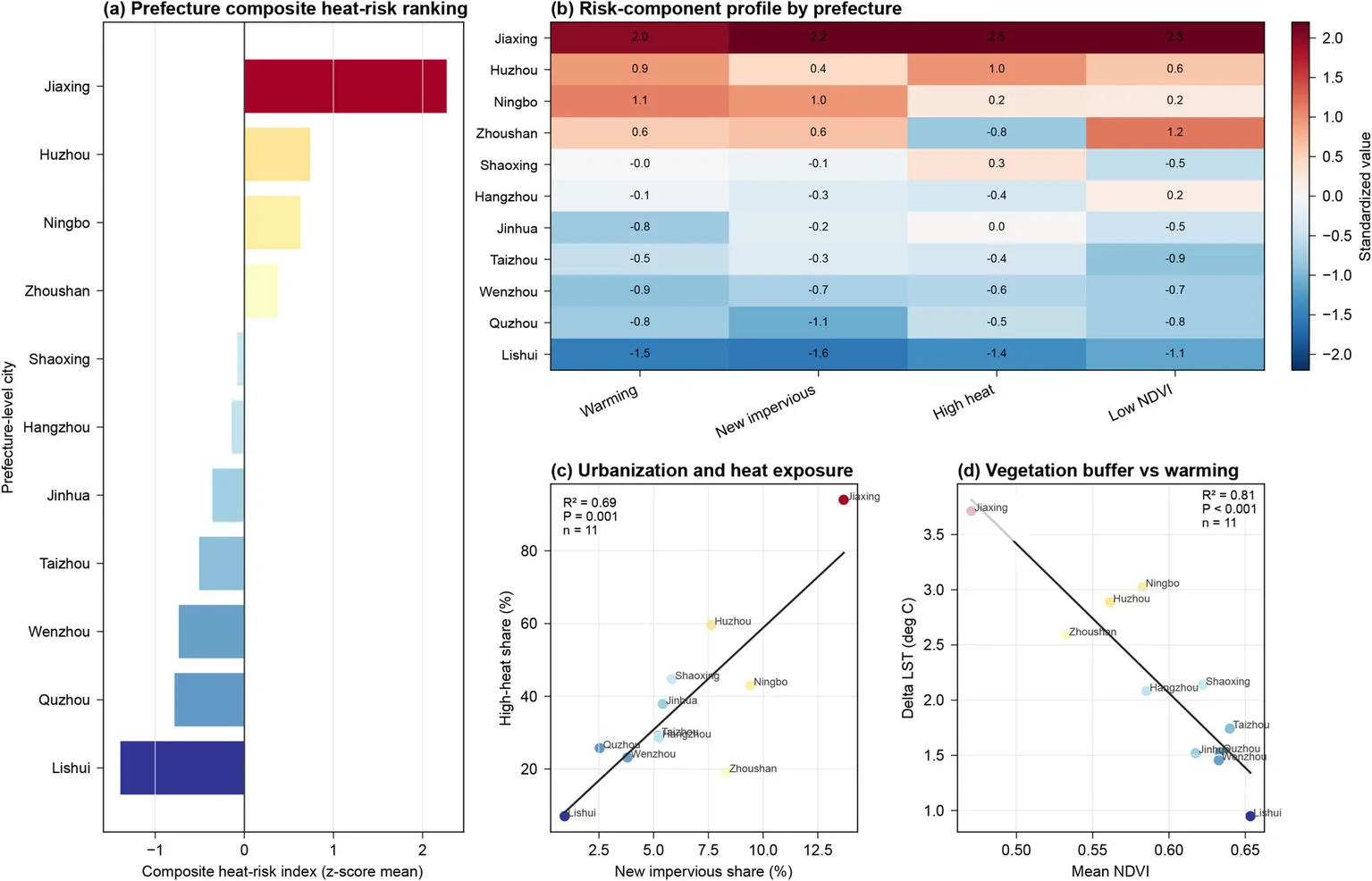
Prefecture-level differentiation of the LST-derived public heat-risk proxy and its associations with urbanization and vegetation cover across the 11 prefecture-level cities of Zhejiang Province. **(a)** Ranking of the composite proxy index; **(b)** standardized contributions of LST warming magnitude, newly added impervious-surface proportion, heat-exposure ratio, and low-NDVI risk to the index; **(c)** relationship between newly added impervious-surface proportion and heat-exposure ratio; and **(d)** relationship between mean NDVI and LST warming magnitude. The composite index is an exploratory, equally weighted LST-derived proxy and does not represent directly observed epidemiological or health outcomes.

The prefecture-level composite heat-risk proxy assessment shows that land-surface heat-exposure risk in the study area was characterized by pronounced spatial differentiation (Figure 8). Among the 11 prefecture-level cities, Jiaxing had the highest composite heat-risk proxy index, reaching 2.28, substantially higher than that of the other cities and indicating the most prominent composite heat-risk characteristics. Huzhou, Ningbo, and Zhoushan had composite heat-risk indices of 0.74, 0.64, and 0.39, respectively, placing them at relatively high levels. By contrast, Lishui, Quzhou, and Wenzhou had lower composite heat-risk indices of −1.40, −0.79, and −0.74, respectively, indicating that these cities were located in relatively low-risk zones within the regional heat-risk pattern (Figure 8a). The risk-composition profiles further reveal clear differences in the sources of heat risk among cities (Figure 8b). Jiaxing showed the highest levels across all four dimensions: warming magnitude, proportion of newly added impervious surface, heat-exposure ratio, and low-NDVI risk, with standardized values of 2.0, 2.2, 2.5, and 2.3, respectively. This suggests that its elevated heat-exposure risk was not attributable to a single factor, but rather reflected the combined effects of rapid warming, strong urban expansion, concentrated heat exposure, and insufficient vegetation buffering. Although Huzhou and Ningbo had lower composite proxy risks than Jiaxing, they still showed positive deviations in warming magnitude and newly added impervious surface. Zhoushan had a relatively low standardized heat-exposure value but a high low-NDVI risk, indicating that its heat-exposure risk may be more closely related to insufficient vegetation cover. In contrast, Lishui had low values in all four risk dimensions, showing strong ecological-buffering characteristics. A significant positive correlation was observed between urban expansion and heat exposure (Figure 8c). Regression results show that the proportion of newly added impervious surface explained 69% of the variation in heat exposure (*R*^2^ = 0.69, *p* = 0.001, *n* = 11), indicating that stronger construction-land expansion at the prefecture-level city scale was associated with a higher heat-exposure ratio. Jiaxing had both the highest proportion of newly added impervious surface and the highest heat-exposure ratio, making it a typical case of urbanization-associated heat-exposure intensification. Meanwhile, vegetation cover had a significant suppressive effect on warming magnitude (Figure 8d). Mean NDVI was strongly and negatively correlated with LST warming magnitude (*R*^2^ = 0.81, *p* < 0.001, *n* = 11), and each 0.1 increase in NDVI corresponded to an average reduction in warming magnitude of approximately 1.35 °C. Overall, urban expansion was significantly associated with elevated heat exposure, whereas higher vegetation cover was associated with weaker surface warming. Together, these two processes shaped the prefecture-level spatial differentiation of the LST-derived heat-risk proxy in the study area.

## 4 Discussion

### 4.1 Land-use change as an important spatial basis for land-surface heat-exposure risk

This study shows that land-use change in the study area from 2000 to 2020 substantially altered the regional land-surface spatial structure and further reshaped the spatial distribution of land-surface heat-exposure risk. During the study period, impervious-surface area increased from 3,750.74 km^2^ to 8,924.35 km^2^, a net increase of 5,173.61 km^2^ and a growth rate of 137.94%. At the same time, forestland and cropland decreased by 2,865.83 km^2^ and 2,201.97 km^2^, respectively. This shift in land-use structure indicates that the study area underwent spatial restructuring characterized by rapid expansion of construction land and contraction of ecological and agricultural spaces. From a heat-exposure perspective, impervious-surface expansion is not merely a change in land-cover type. By replacing evapotranspiring surfaces with heat-storing built materials, it creates spatial conditions associated with elevated summer daytime LST and broader exposure in urbanizing areas. The results show that, among newly added impervious surfaces from 2000 to 2020, cropland contributed 4,584.15 km^2^, accounting for 86.10% of the total source area. This indicates that urban expansion mainly advanced along plains, river valleys, and agricultural spaces around urban fringes. These areas are often also potential spaces for further population and industrial agglomeration. Therefore, the conversion of cropland to impervious surface represents not only the loss of agricultural space but also the potential extension of land-surface heat exposure toward urban peripheries and suburban transition zones. Accordingly, the findings of this study suggest that land-use change is an antecedent process in the formation of land-surface heat-exposure risk (2, 4, 7, 63). These findings identify land-use transition as an antecedent spatial process of land-surface heat exposure. Incorporating land-cover conversion into thermal-environment analysis therefore helps explain how exposure patterns emerge alongside urban expansion and ecological-space contraction, rather than describing temperature outcomes alone (26, 64–66).

### 4.2 Impervious-surface expansion is associated with a shift from localized high temperature to broader exposure risk

From 2001 to 2020, the mean summer daytime LST in the study area increased from 28.59 °C to 30.47 °C, a cumulative increase of 1.89 °C. More importantly, the increase in upper-percentile temperatures was considerably larger than the mean warming: the 95th percentile rose from 31.45 °C to 36.09 °C, an increase of 4.63 °C. This indicates that thermal-environment change in the study area was not a simple upward shift in the mean, but involved a marked expansion of the high-temperature tail. In other words, areas that represented extreme high-temperature conditions in the past had expanded widely by 2020, and thermal-environment pressure was shifting from localized high-temperature patches to broader spatial exposure risk. The comparison among land-change types further reveals the land-use mechanism underlying this process. Newly added impervious surfaces experienced an average warming magnitude of 4.57 °C from 2001 to 2020, higher than stable impervious surfaces, stable cropland, stable water bodies, and stable forestland. At the same time, the heat-exposure ratio of newly added impervious surfaces reached 90.36%, the highest among all land-change types. This result indicates that newly urbanized areas are the most sensitive and concentrated spatial units for the growth of potential public heat risk. By contrast, stable forestland had a warming magnitude of only 1.18 °C and a heat-exposure ratio of only 9.24%, demonstrating a clear buffering effect of stable ecological space in reducing heat risk. This finding has important implications for heat-exposure-risk governance. Traditional thermal-environment studies often focus on high-temperature clustering in urban centers. However, this study shows that urban-fringe areas, transportation corridors, and urban-expansion frontiers where newly added impervious surfaces occur may also become zones of rapidly increasing land-surface heat exposure (52, 67). Because these areas often undergo rapid development while public-service provision lags behind, their heat-risk governance capacity may be weaker than that of mature urban districts. Therefore, under rapid urban-agglomeration expansion, heat-exposure-risk governance should not focus only on existing high-temperature cores, but should also prioritize identifying incremental risk spaces created by newly added impervious surfaces (68–71).

### 4.3 Vegetation-cover change and buffering capacity against land-surface heat-exposure risk

Vegetation cover is an important ecological factor modulating land-surface heat exposure associated with land-use change. The results show a significant negative correlation between NDVI and LST in 2020, with each 0.1 increase in NDVI corresponding to an approximately 1.33 °C decrease in LST. Comparisons across NDVI intervals further indicate that when NDVI increased from 0.2–0.3 to 0.8–0.9, mean LST decreased from 35.52 °C to 28.91 °C, representing a cooling magnitude of 6.61 °C. This demonstrates that improved vegetation cover can substantially reduce summer daytime surface thermal stress and serves as an important natural regulatory mechanism for mitigating LST-derived heat-exposure risk. Temperature differences among land-cover types also reflect the buffering role of vegetation. In 2020, impervious surfaces had the highest mean LST, at 34.86 °C, whereas forestland had the lowest mean LST, at 29.09 °C, a difference of 5.77 °C. More importantly, stable forestland not only had lower contemporaneous temperature but also exhibited the lowest long-term warming magnitude and the lowest heat-exposure ratio. This means that forestland is not only a ‘cool-source space’ in heat-exposure-risk governance, but also critical infrastructure for maintaining the stability of the regional thermal environment. However, the risk-buffering role of vegetation shows clear spatial differences. Although NDVI and LST were significantly negatively correlated, the scatter distributions of different land types did not completely overlap. Water bodies had relatively low NDVI but still lower temperatures than impervious surfaces, indicating that the thermophysical properties of surface types also affect land-surface thermal conditions (58, 71–73). Cropland had moderate NDVI and moderately high LST, suggesting that its thermal-regulation capacity was weaker than that of forestland but clearly stronger than that of construction land. Therefore, from the perspective of heat-exposure-risk governance, increasing green quantity should not be treated as the only strategy. Instead, the land type, vegetation-cover level, and heat-exposure intensity should be used to identify spatial units where ecological intervention is most needed (74–77). In particular, areas where low NDVI, high LST, and newly added impervious surfaces overlap may have higher efficiency for risk reduction through increased vegetation cover.

### 4.4 Prefecture-level heat-risk proxy differentiation reflects the coupling of land-use change and ecological buffering capacity

The prefecture-level city analysis further shows that the LST-derived public heat-risk proxy has pronounced regional differentiation. Jiaxing had the highest composite heat-risk proxy index and showed high values in all four dimensions: warming magnitude, proportion of newly added impervious surface, heat-exposure ratio, and low-NDVI risk. This indicates that its elevated proxy risk was not attributable to a single high-temperature factor, but rather reflected the combined effects of rapid land urbanization, strong heat exposure, and insufficient vegetation buffering. Huzhou, Ningbo, and Zhoushan also exhibited relatively high risks, but their risk compositions differed, indicating that land-surface heat-exposure risk follows different formation pathways across cities. The regression results further support this interpretation. The proportion of newly added impervious surface was significantly and positively correlated with the heat-exposure ratio, with an *R*^2^ of 0.69, indicating that stronger urban expansion was associated with a higher heat-exposure ratio. Mean NDVI was significantly and negatively correlated with LST warming magnitude, with an *R*^2^ of 0.81, indicating that higher vegetation cover was associated with lower warming magnitude. These findings show that the LST-derived heat-risk proxy is not determined simply by climatic background, but is a spatial outcome jointly produced by land-use change and ecological-buffering capacity. This conclusion has direct implications for heat-exposure-risk governance at the urban-agglomeration scale. For high-composite-risk cities such as Jiaxing, priority should be given to controlling newly added impervious-surface expansion, improving the connectivity of urban-fringe green spaces, and reducing heat exposure (37, 78, 79). For cities with prominent low-NDVI risk, strengthening vegetation-buffering capacity should be emphasized. For cities with lower composite risks, such as Lishui and Quzhou, protecting ecological spaces is important to prevent future low-density development from eroding regional cool sources. In other words, heat-exposure-risk governance should not rely on a uniform strategy, but should implement differentiated regulation based on land-use change processes and risk-composition characteristics (8, 80–82).

### 4.5 Planning implications: from land-use regulation to heat-exposure-risk governance

The findings of this study indicate a clear transmission pathway through which land-use change is associated with potential public heat risk: the loss of cropland and forestland provides space for impervious-surface expansion; impervious-surface expansion is associated with increased LST and expanded heat exposure; and vegetation cover and ecological spaces buffer risk during this process. Therefore, territorial spatial planning oriented toward heat-exposure-risk governance should not focus only on controlling the scale of construction land, but should also consider the incremental heat-exposure risk associated with land conversion (28, 41, 83–84). First, newly added impervious surfaces should be adopted as an important indicator for land-surface heat-exposure early warning. Newly added impervious surfaces represent not only construction expansion, but also the formation of potential heat-exposure areas. For urban fringes, industrial parks, transportation corridors, and expanding urban clusters, land-surface heat-exposure assessment should be conducted before development to avoid the continued amplification of heat exposure during the construction of new districts. Second, the risk-buffering functions of cropland and ecological spaces should be strengthened. This study found that cropland was the main source of newly added impervious surfaces, while the thermal-environment condition of cropland itself was substantially better than that of impervious surfaces (13, 34, 66). Therefore, cropland protection has significance not only for food security but also for maintaining low-heat-exposure spaces around cities. Forestland showed the lowest warming magnitude and the lowest heat-exposure ratio, making it the most important ecological barrier in regional heat-exposure-risk governance. Third, vegetation enhancement should be prioritized in high-risk land-conversion areas. Because the cooling effect of vegetation is more pronounced in low-to-medium NDVI intervals, embedding green spaces around newly added impervious surfaces, within stable impervious-surface areas, and in low-NDVI high-temperature exposure zones may be more efficient for heat-exposure reduction than further increasing greenness in already highly vegetated forest areas. Future urban renewal and new-district development should regard blue–green spaces as heat-exposure mitigation infrastructure rather than merely landscape elements (32, 78, 80). In practical planning, the prefecture-level heat-risk proxy index can serve as an early screening tool for identifying cities, urban fringes, and development zones where detailed microclimate simulation, air-temperature monitoring, or population-vulnerability assessment should be prioritized. This is particularly relevant for rapidly urbanizing regions, where integrating heat-exposure indicators into land-use zoning, construction-land approval, ecological-redline protection, and urban-renewal design can help prevent newly developed areas from becoming future heat-exposure hotspots.

### 4.6 Limitations and future research directions

This study analyzed how land-use change is associated with an LST-derived public heat-risk proxy and revealed the relationships among impervious-surface expansion, vegetation cover, and heat exposure. Nevertheless, several limitations remain (18, 43, 81). First, this study used LST to characterize the land-surface thermal environment and identified potential heat-exposure spaces using a high-temperature threshold. LST effectively reflects the influence of land-use change on surface energy processes, but it is not equivalent to near-surface air temperature, human thermal comfort indices such as the Universal Thermal Climate Index (UTCI) or Physiologically Equivalent Temperature (PET), or observed morbidity, mortality, and other human-health outcomes. Therefore, the heat risk discussed in this study should be interpreted as a public heat-risk proxy based on land-surface thermal conditions, rather than as direct evidence of public-health impacts (76). Second, this study did not directly incorporate social-vulnerability variables such as population density, age structure, the distribution of outdoor workers, or accessibility to medical resources. Current risk identification therefore reflects mainly changes in hazard and exposure space. Future studies could further incorporate population exposure, social vulnerability, air-temperature observations, thermal comfort indices, and epidemiological data to construct a more complete public-health risk-assessment framework. Third, this study focused on the summer daytime thermal environment, while nighttime heat stress, heatwave duration, and compound extreme events may have different implications for human exposure. Future research could therefore extend the analysis to multiple periods, multiple seasons, and extreme-event scales. Overall, this study explains the spatial formation of potential public heat risk from the perspective of land-use change, showing that rapid impervious-surface expansion is associated with elevated land-surface heat exposure, while vegetation cover and stable ecological spaces are important foundations for risk buffering. This framework helps advance land-use change research from purely spatial-pattern analysis toward heat-exposure-risk governance, and provides spatial evidence for climate adaptation, territorial spatial optimization, and heat-exposure prevention at the urban-agglomeration scale.

## 5 Conclusion

This study examined how land-use change is associated with land-surface heat-exposure risk in Zhejiang Province by integrating the China Land Cover Dataset (CLCD), Moderate Resolution Imaging Spectroradiometer (MODIS) summer daytime land surface temperature (LST), MODIS Normalized Difference Vegetation Index (NDVI), impervious-surface expansion information, and a prefecture-level public heat-risk proxy index. The strongest empirical finding is that impervious-surface expansion, especially newly added impervious surfaces, was consistently associated with higher warming magnitude and heat exposure. From 2000 to 2020, impervious surfaces increased from 3,750.74 km^2^ to 8,924.35 km^2^, while forestland and cropland decreased by 2,865.83 km^2^ and 2,201.97 km^2^, respectively. More than 85% of newly added impervious surfaces originated from cropland, indicating that the conversion of agricultural space into construction land was the dominant land-transition pathway linked to expanded heat exposure. Summer daytime LST also showed both overall warming and high-temperature-tail expansion: the mean LST increased from 28.59 °C in 2001 to 30.47 °C in 2020, while the 95th percentile increased from 31.45 °C to 36.09 °C. Among land-change groups, newly added impervious surfaces had the highest warming magnitude (4.57 °C) and the highest heat-exposure ratio (90.36%), whereas stable forestland had the lowest warming magnitude (1.18 °C) and heat-exposure ratio (9.24%). At the prefecture-level city scale, newly added impervious-surface proportion was positively associated with heat exposure (*R*^2^ = 0.69), while mean NDVI was negatively associated with LST warming magnitude (*R*^2^ = 0.81). These results indicate that land-urbanization intensity and ecological-buffering capacity jointly shape the spatial pattern of the LST-derived public heat-risk proxy. The findings can inform urban planning and heat mitigation in three ways: limiting heat-intensive conversion from cropland or forestland to impervious surface, protecting cropland and forestland as thermal-buffering spaces, and strengthening connected blue–green infrastructure in newly urbanized, low-NDVI, and high-exposure areas. Incorporating heat-exposure screening into land-use approvals, urban expansion boundaries, ecological protection redlines, and new-district design would help reduce the formation of future heat-exposure hotspots. Because this study is based on LST-derived exposure indicators rather than observed health records, the findings should be interpreted as evidence of land-surface heat-exposure risk, while the prefecture-level composite index represents an LST-derived public heat-risk proxy rather than a direct estimate of morbidity, mortality, or other health outcomes.

## Data Availability

The raw data supporting the conclusions of this article will be made available by the authors, without undue reservation.
